# Search of Neuroprotective Polyphenols Using the “Overlay” Isolation Method

**DOI:** 10.3390/molecules23081840

**Published:** 2018-07-24

**Authors:** Hiroshi Sakagami, Haixia Shi, Kenjiro Bandow, Mineko Tomomura, Akito Tomomura, Misaki Horiuchi, Tomohiro Fujisawa, Takaaki Oizumi

**Affiliations:** 1 Meikai University Research Institute of Odontology (M-RIO), 1-1 Keyakidai, Sakado, Saitama 350-0283, Japan; 2 Department of Traditional Chinese Medicine, Shanghai Ninth People’s Hospital, Shanghai Jiaotong University School of Medicine, Shanghai 201900, China; 3 Divisions of Biochemistry, Department of Oral Biology and Tissue Engineering, Meikai University School of Dentistry, Saitama 350-0283, Japan; kbando@dent.meikai.ac.jp (K.B.); mineko-t@dent.meikai.ac.jp (M.T.); atomomu@dent.meikai.ac.jp (A.T.); 4 Daiwa Biological Research Institute Co., Ltd., Kanagawa 213-0012, Japan; m_horiuchi@daiwaseibutsu.co.jp (M.H.); t_fujisawa@daiwaseibutsu.co.jp (T.F.); takaakio@daiwaseibutsu.co.jp (T.O.)

**Keywords:** neuroprotection, PC12, NGF, differentiation, amyloid-β peptide, taxanes, hormesis, polyphenol, bamboo leaf extract, overlay method

## Abstract

Previous studies of the neuroprotective activity of polyphenols have used ununiform culture systems, making it difficult to compare their neuroprotective potency. We have established a new and simple method for preparing differentiated PC12 cells by removing the toxic coating step. Cells were induced to differentiate with the nerve growth factor (NGF) in a serum-free medium, without a medium change, but with a one-time overlay supplementation of NGF. The optimal inoculation density of the cells was 6–12 × 10^3^ cells/cm^2^, and the presence of serum inhibited the differentiation. Neuroprotective activity could be quantified by the specific index (SI) value, that is, the ratio of the 50% cytotoxic concentration to the 50% effective concentration. Alkaline extract from the leaves of *Sasa senanensis* Rehder (SE), having had hormetic growth stimulation, showed the highest SI value, followed by epigallocatechin gallate. The SI value of curcumin and resveratrol was much lower. This simple overly method, that can prepare massive differentiated neuronal cells, may be applicable for the study of the differentiation-associated changes in intracellular metabolites, and the interaction between neuronal cells and physiological factors.

## 1. Introduction

Improvement in the daily nutritional supply and the living environment resulted in the prolongation of our life span, but necessarily increased the number of elderly populations having cognitive diseases [1,2,3]. Alzheimer’s disease, the most common form of dementia, is characterized by the accumulation of amyloid-β (Aβ) in the brain. Since the neurotoxicity of Aβ has been well established [4,5], preventing the accumulation [6] and early oligomerization [7] of Aβ may be a promising cognitive behavioral therapy. 

Platinum drugs such as cisplatin, carboplatin and oxaliplatin have been important parts of combination chemotherapy regimens to treat different types of solid tumors, but they can cause serious neurotoxicity in the dorsal root ganglion by the formation of adducts to DNA [8]. Taxanes, such as paclitaxel and docetaxel, induce microtubule assembly, mitotic arrest, and finally apoptosis in cancer cells. However, they often induce painful peripheral neurotoxicity during treatment [9]. Patients who received both platinum and taxane treatment showed aggravated neuropathy [10].

Polyphenols—defined as substances that possess an aromatic ring bearing one or more hydroxyl substituents—have been reported to show neuroprotective activity. Polyphenols are roughly classified into the following three groups: Tannins; flavonoids; and, lignin-carbohydrate complexes (LCC) [11]. Tannins are classified into two large groups: Hydrolysable tannins (in which a polyalcohol is esterified with a polyphenolic carboxylic acid such as a galloyl, hexahydroxydiphenoyl, valoneoyl, or dehydrohexahydroxydiphenoyl group); and, condensed tannins (composed of flavan units, mostly catechin, epicatechin, or their analogs, condensed with each other via carbon–carbon bonds) [12]. Flavonoids are secondary metabolites synthesized from chalcones [13] and are categorized into flavonols, flavones, flavanones, isoflavones, pterocarpan and coumestan. Resveratrol is classified as a stilbenoid. Lignins are formed through phenolic oxidative coupling processes. Lignin macromolecules are formed by the dehydrogenative polymerization of three monolignols: *p*-coumaryl, *p*-conifery, and sinapyl alcohols. Some polysaccharides in the cell walls of lignified plants are linked to lignin, and recover as lignin-carbohydrate complex—after extraction with alkaline solution [14].

Previous studies have mostly used low-molecular-weight polyphenols, such as flavonoids and tannins, which manifest various health-promoting activities (antioxidant, anti-inflammatory, and antibacterial activity). This was due to the recent development of separation technology for elucidating their chemical structure [15,16]. On the other hand, the search for neuroprotective, high-molecular substances, such as lignin-carbohydrate complex, have been delayed due to their amorphous structures. They did, however, show prominent anti-HIV activity [17]. 

Most previous studies of neuroprotection have used rat pheochromocytoma 12 (PC12) [18] and human SH-SY5Y neuroblastoma cell lines [19,20], since these cell lines differentiate into neuronal cells with elongated neurites upon treatment with nerve growth factor (NGF) or retinoic acid.

However, there was no uniformity in the culture condition of PC12 and SH-SY5Y cells in previous investigations. The culture media used was: Dulbecco’s modified Eagle’s medium (DMEM); the minimum essential medium (MEM); RPMI1640; a mixture of DMEM and Ham′s F12 (1:1); and, non-essential amino acids (NEAA), which were supplemented with fetal bovine serum (FBS), alone or together with, horse serum (HS) (column A in Table 1) [21,22,23,24,25,26,27,28,29,30,31]. Differentiated and undifferentiated cells (column B), inoculated at different cell densities, were exposed to considerably different concentrations of neurotoxic agents (column C) in uncoated, collagen, or poly-lysine-coated plates (Table 1). Most importantly, previous investigators have not presented the chemotherapeutic index (safety margin). Therefore, it was difficult to compare with previous data on the neuroprotective activity of polyphenols. 

In order to investigate the interaction between neuroprotective substances and cells, it is best to use the medium that has the simplest components. We have recently reported that the addition of Ham’s F-12 and non-essential amino acids did not increase, but rather, reduced the growth and amino acid consumption of both PC12 and SH-SY5Y cells [32]. During neuronal differentiation induced by NGF, a medium change (that removes numerous neurotrophic factors released from differentiating cells) at an early stage resulted in the poor attachment and low recovery of differentiated cells. If a fresh NGF-containing medium was supplemented by an overlay at the middle stage (without a medium change), comparable numbers of differentiated cells could be harvested after six or seven days, regardless of the types of plates, either non-coated or coated with collagen I or IV. To our surprise, due to its toxicity, the use of poly-lysine coated plates significantly reduced the yield of differentiated cells [33]. Based on these results, DMEM, supplemented with NGF, was adopted as a regular differentiation induction medium, which was added to the culture plates that were not coated with collagen I, IV, or poly-lysine.

Using PC12 cells prepared by the overlay method, we have re-investigated the neuroprotective activity of various polyphenols. This was compared with that of plant extracts and antioxidants, based on the specific index (SI) (defined as the ratio of 50% cytotoxic concentration (CC_50_) to 50% protective concentration (EC_50_)).

## 2. Results

### 2.1. Optimal Concentration of Ngf and Fbs for the Induction of PC12 Cell Differentiation

**Figure 1:**
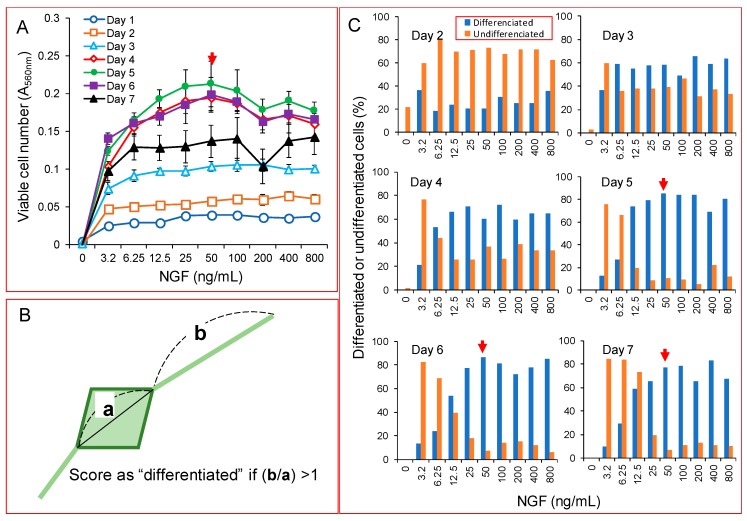
Stimulation of the growth and differentiation of PC12 cells by NGF. Cells were inoculated at 2 × 10^3^/96-microwell plate (6.25 × 10^4^/cm^2^). After 24 h, the medium was replaced with a serum-free medium containing the indicated concentrations of NGF. At day three, additional NGF was supplemented on top of the medium. Viable cell number (**A**), and the percentage of differentiated cells, defined as in (**B**), and undifferentiated cells (counted after subtraction of differentiated cells and dead cells) (**C**), was then determined. In (**B**), (a) is the longest diameter of the cells, and (b) is the length of extended neurites. Each value represents the mean ± S.D. of 6 determinations.

We first confirmed that NGF stimulated the growth of PC12 cells at the optimal concentration of 50 ng/mL in our assay system. The cell number increased up to day five, and thereafter, slightly declined, possibly due to it reaching the terminal differentiation (Figure 1A). NGF also stimulated the formation of neurite—the marker of neuronal differentiation. When differentiated cells were defined as the cells in which the extended neurite exceeds the longest diameter of each cell (Figure 1B), the percentage of differentiated cells reached a plateau at day five, with a maximum of 50 ng/mL NGF (Figure 1C).

### 2.2. FBS Inhibited the Differentiation of PC12 Cells

**Figure 2:**
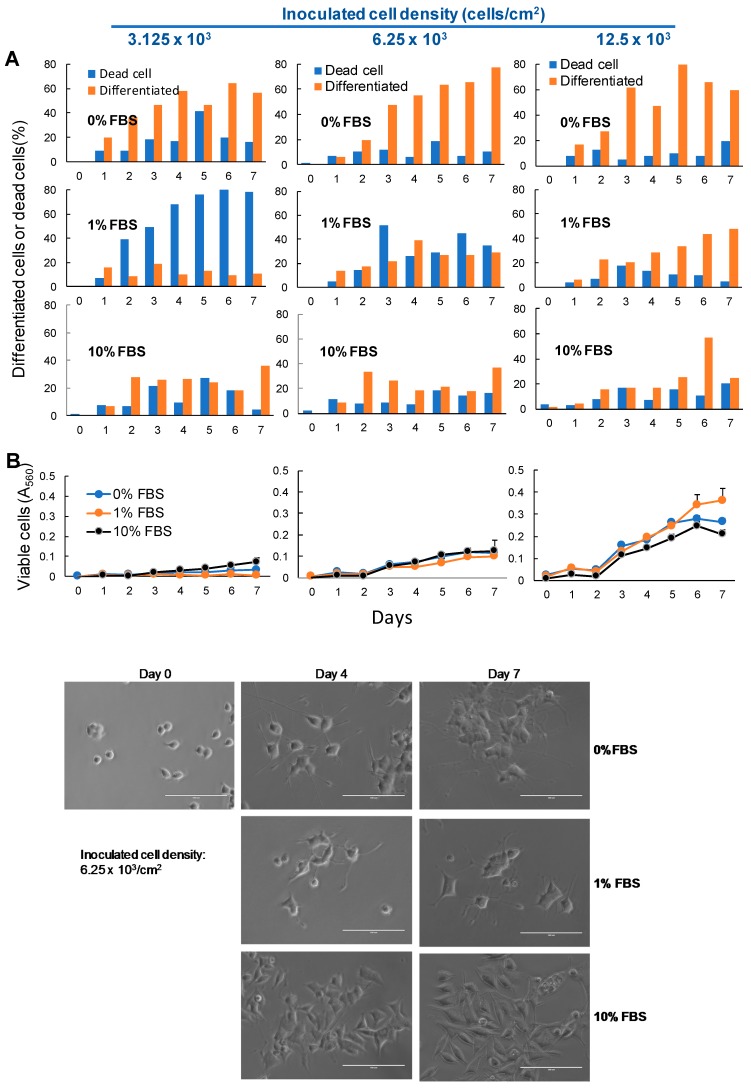
FBS reduced the neuronal differentiation of PC12 cells. PC12 cells were seeded at 3.125, 6.25 and 12.5 × 10^3^/cm^2^ into a 96-microwell plate. After 24 h, cells were replaced with DMEM supplemented with 0%, 1% or 10% FBS containing 50 ng/mL NGF, and were incubated for zero, four or seven days, with a one-time supplementation of NGF overlay at day three. The percentage of differentiated cells and undifferentiated cells (defined in the legend of Figure 1) (**A**), viable cell number (**B**) and morphological changes were monitored. To score the number of differentiated cells, the morphology of more than 100 cells were observed under the light microscope.

When PC12 cells were cultured in a serum-free medium, neuronal differentiation reached a maximum level. When inoculation of cell density was reduced to 3.125 × 10^3^/cm^2^, the incidence of cell death slightly increased (Figure 2), which suggests the importance of continuous nutritional supply from neighboring cells. It was unexpected that the maximum differentiation can be achieved in the absence of FBS, and the addition of 1% or 10% FBS inhibited the differentiation induction. Thus, the optimal inoculation of cell density was determined to be between 6 and 12 × 10^3^/cm^2^ in the absence of serum. 

### 2.3. Exploration of the Overlay Method

**Figure 3:**
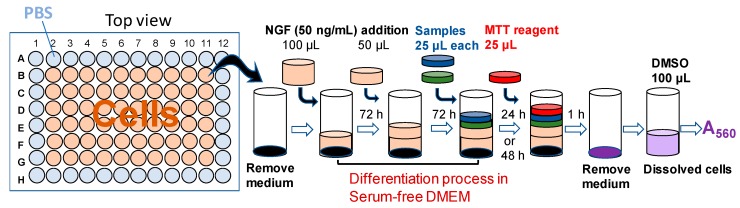
Schematic diagram of overlay method.

Based on this experimental data, we explored the overlay method to isolate differentiating PC12 cells. PC12 cells were incubated for a total of six or seven days in serum-free DMEM, containing 50 ng/mL NGF, with one more time overlay of NGF (Figure 3). The differentiated cells were well attached to the plate and were not detached by gentle pipetting.

### 2.4. Apoptosis-Inducing Activity of Neurotoxic Agents

**Figure 4:**
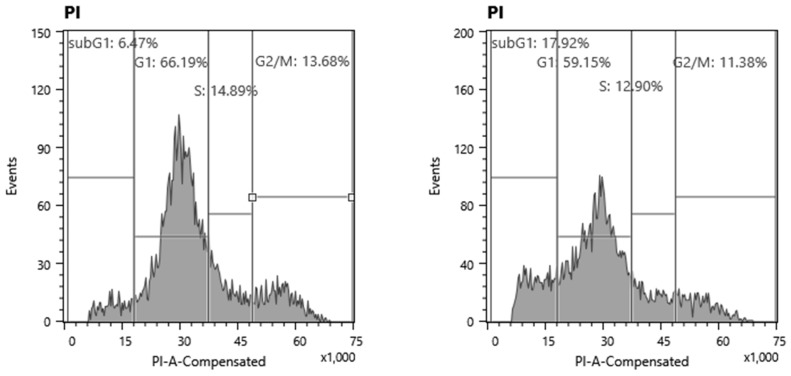
Cell cycle analysis of differentiated PC12 cells (day three) after treatment with neurotoxic agents. Each value represents the mean ± S.D. of triplicate assays.

We have previously reported that cisplatin showed potent cytotoxicity against differentiated PC12 cells [34], while taxanes (paclitaxel, docetaxel) and amyloid peptides (Aβ_1–42_, Aβ_25–35_) showed cytostatic effects [34,35]. A cell sorter analysis demonstrated that cisplatin induced the apoptosis, characterized by the accumulation of the subG_1_ population (Figure 4). On the other hand, paclitaxel, Aβ_1–42_ and Aβ_25–35_ reduced the population of S-phase cells—reflecting their cytostatic growth inhibition—and accumulated the G2 + M phase cells, in accordance with the reported mitotic arrest by taxanes [36,37]. It was unexpected that differentiated cells were resistant to actinomycin D—a popular apoptosis inducer for various cancer cell lines.

### 2.5. Neuroprotective Effects of Polyphenols

**Figure 5:**
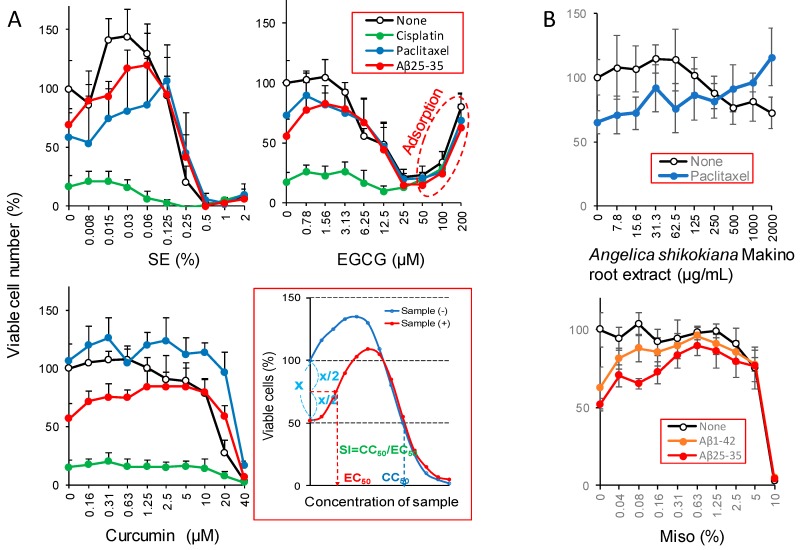
Neuroprotective effects of various polyphenols and plant extracts. Concentration of neurotoxic agents are: Aβ_25–35_ (500 nM); cisplatin (50 μM); and, paclitaxel (50 nM).

Alkaline extract from the leaves of *Sasa senanensis* Rehder (SE) protected the cytotoxicity induced by paclitaxel and Aβ_25–35_, but not the cytotoxicity induced by cisplatin. Epigallocatechin gallate (EGCG) and curcumin showed some protective activity when induced by Aβ_25–35_ (Figure 5A). 

Table 2 shows the SI (=CC_50_/EC_50_) value of polyphenols, plant extracts and antioxidants, calculated using the data from our original papers [33,35,38,39] and the present study (Table 2). The higher the SI value, the stronger the neuroprotective activity would be expected.

#### 2.5.1. Neuroprotective Activity against Undifferentiated PC12 and SH-SY5Y Cells (Exp. 1)

The SE showed the highest neuroprotective activity (SI = 37.2, 141.4, >108, <1, <1, 7.1, 5.9, 5.4), followed by EGCG (SI = 6.1, <1, <1, <1). However, resveratrol (SI = <1, ><1, <1, <1), curcumin (SI = <1, <1, <1, <1) and *p*-coumaric acid (SI = ><1, ><1, ><1, ><1) showed no apparent neuroprotective activity [33].

#### 2.5.2. Protective Activity against Differentiated PC12 Cells (Exp. 2)

SE showed the highest neuroprotective activity (SI = 45.8, 73.3, 40.2, 7.7, <1), followed by EGCG (SI = 10.7, 15.1, <1), and curcumin (SI = 17.3, <1). Resveratrol did not show any apparent protective activity (SI < 1). None of these substances were protective against cisplatin-induced neurotoxicity.

#### 2.5.3. Neuroprotective Activity of Plant Extracts (Exp. 3)

*Angelica shikokiana* Makino extract [38] and the hot water extract of Miso—a traditional Japanese fermented food that has supported our diet for many years [39]—showed neuroprotective effects against paclitaxel and amyloid peptides (Figure 5B). Especially, a significant (*p* < 0.05) growth stimulation effect of Miso on Aβ_1–42_ and Aβ_25–35_-treated cells was observed above 0.04%.

### 2.6. Growth Stimulation by SE

**Figure 6:**
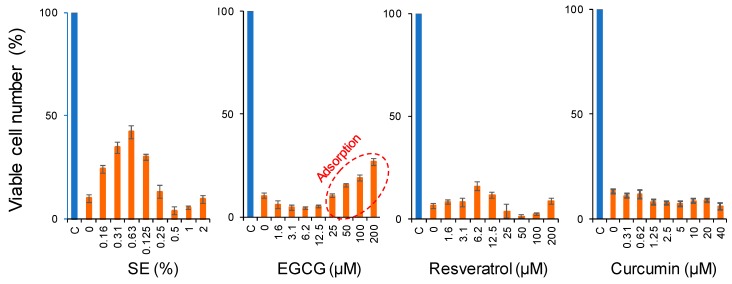
Growth stimulation activity of various polyphenols. PC12 cells were cultured for 24 h in DMEM, supplemented with 10% FBS (indicated by C (control) in blue), or without serum (red) in the presence of indicated concentrations of test samples. The viable cell number was then measured by 3-(4,5-dimethylthiazol-2-yl)-2,5-diphenyltetrazolium bromide (MTT) reagent, and expressed as a percentage of control (C). Each value represents the mean ± S.D. of six determinants.

There was a possibility that neuroprotective effects may be related to the growth stimulation against PC12 cells. To test this possibility, PC12 cells were cultured for 24 h in a serum-free medium containing various sample concentrations. The removal of serum from the cultured medium reduced the growth potential of PC12 cells to 10% of the control level (Figure 6). By the addition of SE, the viability returned back to 40% of the control level, whereas the growth stimulation effects of EGCG, resveratrol, and curcumin was much less (Figure 6).

## 3. Discussion

We have explored a new and simple method that enabled us to prepare differentiated neuronal cells by the repeated overlay of the NGF-containing medium without a medium change, omitting the use of expensive collagen-coated plates, or the toxic coating step. Using the overlay method, we re-investigated the neuroprotective activity of polyphenols.

SE, a group of three over-the-counter drugs, showed the highest neuroprotective activity against both undifferentiated and differentiated PC12 cells, as well as undifferentiated SH-SY5Y cells. This seems to be linked to growth stimulation at lower doses, known as hormesis [40], since we have experienced similar growth stimulation of SE towards human gingival epithelial fibroblast (HGEP) [41] and PC12 cells [33]. SE showed prominent anti-HIV [42] and anti-UV activity [43], like the lignin-carbohydrate complexes extracted with alkaline solution. The present study further adds that SE showed potent neuroprotective activity. SE contains the lignin-carbohydrate complex and the various degradation products, and thus not a purified material. The lignin-carbohydrate complex showed poor bioavailability [44], and its biological activity might be mediated through one of the pattern-recognition receptors (dectin-2) in the mucosa of oral cavities [45] and intestinal duct. We found that *p*-coumaric acid, one of the lignin precursors present in SE (unpublished data), has no neuroprotective activity. Further fractionation of SE is necessary to identify the neuroprotective substances present in SE.

EGCG, a main component of green tea, showed some neuroprotective activity. We found that higher concentrations of EGCG led to false-positive coloring with MTT reagent—indicated by the red circle in Figure 5 and Figure 6—and were possibly due to the non-specific binding of tannin to protein [46,47]. Neuroprotective effects of EGCG may be mediated through cell-surface receptors [48] or by the direct binding to neurotoxic agents.

Curcumin showed some neuroprotective activity against differentiated PC12 cells, but not against undifferentiated cells. The fluctuation in the SI value of this compound may be due to its potent cytotoxicity, narrowing the chemotherapeutic range.

Food polyphenols, at low and nontoxic concentrations, slow down the progression of neurodegenerative diseases and therefore may be a promising approach [49]. It was unexpected that Miso extract also showed neuroprotective (Figure 5B) and growth promotion activity [38], further substantiating the possible link between neuroprotective activity and hormesis. Since Miso is a daily food, it is very much meaningful to identify the active principle for the future research aiming at alleviating the neuronal diseases.

As a mechanism of neuronal protection, activation of the NF-E2 related factor 2 (Nrf2) pathway and the consequent upregulation of detoxification enzymes, such as heme-oxygenase-1 (HO-1), have been suggested [50,51]. However, we have recently reported that the neuroprotective activity of four antioxidants: Docosahexaenoic acid; acetyl-l-carnitine hydrochloride; *N*-acetyl-l-cysteine; and, sodium ascorbate, only partially protected PC12 cells from paclitaxel-induced toxicity [35]. Furthermore, it has recently been reported that neuronal protection caused by blackberry polyphenols is produced by mechanisms other than modulating reactive oxygen species (ROS) levels [52]. It is possible that both oxidative and other mechanisms, such as: Autophagy [21]; heat shock protein (HSP)-70 [22]; JAK2/STAT5/Bcl-xL [24]; phosphor HSP-20 [28]; amyloid disaggregation [29]; and, the overexpression of the *KiSS* gene [30], may be involved in the induction of neurotoxicity (column D in Table 1). 

We have recently manufactured highly tumor-specific derivatives from the chromone core structure found in flavones, isoflavones and 2-styrylchromones, and discovered that their tumor-specificity was correlated with molecular shape and hydrophobicity [53,54]. By analogy, for searching new neuroprotective substances, it may be one way to introduce various functional groups to the core substance present in the natural kingdom, repeat the synthesis, and verify the process using QSAR analysis. 

## 4. Materials and Methods 

### 4.1. Materials

The following chemicals and reagents were obtained from the indicated companies: DMEM; human recombinant NGF; paclitaxel; propidium iodide (PI); actinomycin D (Act. D); 4% paraformaldehyde phosphate buffer solution; dimethyl sulfoxide (DMSO) (Wako Pure Chemical Ind., Ltd., Osaka, Japan); Nonidet P-40 (NP-40) (Nakalai Tesque Inc., Kyoto, Japan); FBS; 3-(4,5-dimethylthiazol-2-yl)-2; 5-diphenyltetrazolium bromide (MTT) (Sigma-Aldrich Inc., St. Louis, MO, USA); Aβ_1–42_ and Aβ_25–35_ (Cosmo Bio Co., Ltd., Tokyo, Japan); NGF (dissolved in water at 0.5 mg/mL); Aβ_1–42_ (dissolved in DMSO at 1 mM); and, Aβ_25–35_ (dissolved in water at 0.1 mM) were frozen at −20 °C. The following 96-microwell plates were purchased from TPP (Techno Plastic Products AG, Trasadingen, Switzerland).

### 4.2. Cell Culture 

PC12 cells were purchased from Riken Cell Bank (Tsukuba, Japan). These cells were cultured in DMEM and were supplemented with 10% FBS, 100 units/mL, penicillin G and 100 µg/mL streptomycin under a humidified 5% CO_2_ atmosphere. 

### 4.3. Determination of Viable Cell Numbers 

To the culture medium, a fresh medium containing 3-(4,5-dimethylthiazol-2-yl)-2,5-diphenyltetrazolium bromide (MTT) (final concentration: 0.1 mg/mL) with (for differentiated cells) or without (for non-differentiated cells) of 50 ng/mL NGF. The cells were incubated for 1 h, and the formazan precipitate was dissolved in DMSO to measure their absorbance at 560 nm with a plate reader (Infinite F 50 R, TECAN, Kawasaki, Japan). 

### 4.4. Induction of Differentiation Toward Neurons 

For the induction of differentiation, PC12 cells were incubated for the indicated times with a differentiating inducing medium (DMEM, supplemented with 0% or 1% FBS, 50 ng/mL NGF and antibiotics). Differentiated cells were defined as the cells in which the extended neuritis exceeded the longest diameter of each cell, assessed under the light microscope (EVOS FL, ThermoFisher Scientific, Waltham, MA, USA) (Figure 1B).

### 4.5. Neuroprotection Assay

Undifferentiated (PC12, SH-SY5Y) and differentiated PC12 cells were pretreated for 1 h with test samples, and then neurotoxic agents were added (0.3 μM, Aβ_1–42_, 2 μM Aβ_25–35_, 50 μM cisplatin, 0.4 μM paclitaxel). After treatment for 24 or 48 h, viable cell numbers were determined, as described above. The 50% cytotoxic concentration (CC_50_) was determined from the neurotoxicant-free culture, and the 50% effective concentration (EC_50_) that reduced the neurotoxicant-induced cytotoxicity by 50% was calculated (Figure 5). The protective effect was quantified by the SI, using the following formula: SI = CC_50_/EC_50_ (Figure 5).

### 4.6. Cell Cycle Analysis

Cells (approximately 10^6^) were harvested, fixed with a 1% paraformaldehyde in phosphate-buffered saline without calcium or magnesium ions [PBS(−)]. Fixed cells were washed twice with PBS(−), and then treated for 30 min with 400 μL of 0.2 mg/mL RNase A (preheated for 10 min at 100 °C to deactivate DNase) to degrade RNA. Cells were then washed twice with PBS(−) and stained for 15 min with 0.005% propidium iodide (PI) in the presence of 0.01% NP-40 in PBS(−), which prevents cell aggregation. After filtering through Falcon^®^ cell strainers (40 μM) (Corning Inc., Corning, NY, USA) to remove aggregated cells, PI-stained cells were subjected to a cell sorter (SH800 Series, SONY Imaging Products and Solutions Inc., Kanagawa, Japan). Cell cycle analysis was performed with the Cell Sorter Software version 2.1.2 (SONY Imaging Products and Solution Inc., Kanagawa, Japan).

### 4.7. Statistical Treatment

Experimental values are expressed as the mean ± standard deviation (SD) of triplicate or quadruplicate samples. Statistical analysis was performed using Student’s *t*-test. A *p*-value of <0.05 was considered to be significant.

## 5. Conclusions

The present study demonstrated that plant extracts, having hormetic growth stimulation, showed higher neuroprotective activity than lower molecular weight polyphenols. The overlay method, that can prepare massive differentiated neuronal cells, may be applicable for the study of differentiation-associated changes in intracellular metabolites by metabolomics, and the interaction between neuronal cells and physiological factors. 

## Figures and Tables

**Table:** 

		Distribution of Each Cell Cycle Phase (%)											
		subG1			G1			S			G2 + M		
Control		8.1	±	1.4	67.6	±	1.2	14.2	±	0.6	11.3	±	2.1
Cisplatin	10 µM	11.8	±	0.8	69.6	±	1.0	12.9	±	0.5	7.0	±	0.2
	30 µM	13.3	±	0.6	69.7	±	0.3	11.9	±	0.6	6.3	±	0.4
	100 µM	18.3	±	0.3	59.7	±	0.7	12.3	±	0.5	11.0	±	0.5
Paclitaxel	0.1 µM	7.1	±	0.5	67.4	±	0.6	9.1	±	0.3	17.3	±	0.9
	0.3 µM	7.8	±	0.4	66.0	±	0.6	11.4	±	2.2	15.8	±	2.2
	1 µM	8.6	±	0.3	66.3	±	0.9	9.2	±	0.7	16.7	±	0.7
Aβ_1–42_	36 nM	8.6	±	0.4	68.5	±	1.1	8.9	±	0.7	14.8	±	0.9
	100 nM	6.6	±	0.4	68.6	±	1.0	9.0	±	0.4	16.6	±	0.9
	360 nM	8.6	±	0.3	68.4	±	0.3	8.3	±	0.0	15.4	±	0.3
Aβ_25–35_	143 nM	8.7	±	1.4	67.3	±	1.3	9.3	±	0.4	15.7	±	0.7
	429 nM	6.6	±	1.2	67.4	±	0.8	9.8	±	0.6	16.9	±	0.8
	1430 nM	7.5	±	1.0	67.7	±	0.6	9.3	±	0.5	16.4	±	0.5
Actinomycin D	1 µM	3.3	±	0.2	66.4	±	1.2	11.4	±	0.4	20.8	±	0.4
	2 µM	3.6	±	1.3	66.0	±	1.3	15.5	±	1.4	16.3	±	1.3

**Table 1:** Culture system for PC12 cells used by previous researchers.

Cell	Culture Medium	Differentiation-Induction Medium	Cytotoxicity Induced by	Rescue System	Ref
	(A)	(B)	(C)	(D)	
PC12	RPMI1640 + 10%FBS + 5%HS	NGF (50 ng/mL) in serum-free RPMI1640 for 7 days	Aβ_25–35_ (5–10 μM)	Autophagy	[21]
PC12	RPMI1640 + 10%FBS + 5%HS	NGF (25 ng/mL) in RPMI1640 + 10%FBS + 5%HS in collagen-coated dish for 2 days	Aβ_25–35_ (10 μM)	HSP-70	[22]
PC12	RPMI1640 + 5%FBS + 10%HS	No treatment	Aβ_1–42_ (75 μM)	ROS reduction	[23]
PC12	DMEM + 10%FBS	No treatment	Aβ_25–35_ (20 μM)	JAK2/STAT5/Bcl-xL	[24]
PC12	DMEM + 10%FBS	No treatment	Aβ_25–35_ (20 μM)		[25]
SH-SY5Y	DMEM + 10%FBS	No treatment	Aβ_1–42_ (0.5 μM)	HO-1 CO	[26]
SH-SY5Y	DMEM/Ham’s F-12 (1:1) + 10%FBS	All-trans-retinoic acid (10 μM) + 3%FBS for 7–8 days	Aβ_25–35_ (20 μM)		[27]
SH-SY5Y	DMEM/Ham’s F-12 (1:1) + 10%FBS	No treatment	Aβ_1–42_ (1–50 μM)	Phospho HSP-20	[28]
SH-SY5Y	MEM/Ham’s F-12 (1:1) + 10%FBS	No treatment	Aβ_1–42_ (20 μM)	Amyloid disaggregation	[29]
SH-SY5Y	DMEM/Ham’s F-12 (1:1) + 10%FBS + 1%NEAA	No treatment	Aβ_1–40_ (10 μM)	KiSS overexpression	[30]
PC12	DMEM/Ham’s F-12 (1:1) + 5%FBS + 10%HS	NGF (25 ng/mL) in RPMI1640 + 10%FBS + 5%HS in collagen-coated dish			[31]

FBS, fetal bovine serum; HS, horse serum; NEAA, non-essential amino acids.

**Table 2:** Neuroprotective activity of polyphenols and plant extracts.

	Target Cell		FBS	Toxicant	Protective Substance	CC_50_		EC_50_		SI	Ref
Exp. 1	<Undifferentiated cell system>										
	SH-SY5Y	Day 0	10%	Aβ_1–42_	SE	4.19	%	0.11	%	37.2	[33]
				Aβ_1–42_	SE	2.22	%	0.016	%	141.4	[33]
				Aβ_1–42_	EGCG	66	μM	>400	μM	6.1	[33]
				Aβ_1–42_	Resveratrol	387	μM	>400	μM	<1	[33]
				Aβ_1–42_	Curcumin	46	μM	>200	μM	<1	
				Aβ_1–42_	*p*-Coumaric acid	>400	μM	>400	μM	><1	
			10%	Aβ_25-35_	SE	2.69	%	<0.21	%	>108	[33]
				Aβ_25–35_	SE	2.22	%	>3.13	%	<1	[33]
				Aβ_25–35_	EGCG	47.8	μM	>400	μM	<1	[33]
				Aβ_25-35_	Resveratrol	>400	μM	>400	μM	><1	[33]
				Aβ_25–35_	Curcumin	17.4	μM	>200	μM	<1	
				Aβ_25–35_	*p*-Coumaric acid	>400	μM	>400	μM	><1	
	PC12	Day 0	10%	Aβ_1–42_	SE	1.38	%	>1.56	%	<1	[33]
				Aβ_1–42_	SE	0.71	%	0.1	%	7.1	[33]
				Aβ_1–42_	EGCG	40.1	μM	>400	μM	<1	[33]
				Aβ_1–42_	Resveratrol	326	μM	>400	μM	<1	[33]
				Aβ_1-42_	Curcumin	19.1	μM	>200	μM	<1	
				Aβ_1–42_	*p*-Coumaric acid	>400	μM	>400	μM	><1	
			10%	Aβ_25–35_	SE	1.15	%	0.21	%	5.9	[33]
				Aβ_25–35_	SE	0.71	%	0.13	%	5.4	[33]
				Aβ_25–35_	EGCG	33.1	μM	>400	μM	<1	[33]
				Aβ_25–35_	Resveratrol	370	μM	>400	μM	<1	[33]
				Aβ_25–35_	Curcumin	60.2	μM	>200	μM	<1	
				Aβ_25–35_	*p*-Coumaric acid	>400	μM	>400	μM	><1	
Exp. 2	<Differentiated cell system>										
	PC12	Day 7	1%	Aβ_1–42_	SE	0.55	%	0.012	%	45.8	[33]
			1%	Aβ_25–35_	SE	0.55	%	0.0075	%	73.3	[33]
	PC12	Day 6	0%	Aβ_25–35_	SE	0.2	%	0.005	%	40.2	
			0%	Paclitaxel	SE	0.2	%	0.026	%	7.7	
			0%	Cisplatin	SE	0.2	%	>2	%	<1	
			0%	Aβ_25–35_	EGCG	8.33	μM	0.78	μM	10.7	
			0%	Paclitaxel	EGCG	8.33	μM	0.55	μM	15.1	
			0%	Cisplatin	EGCG	8.33	μM	>25	μM	<1	
			0%	Cisplatin	Resveratrol	60.4	μM	>200	μM	<1	
			0%	Aβ_25–35_	Curcumin	15.7	μM	0.91	μM	17.3	
			0%	Cisplatin	Curcumin	15.7	μM	>200	μM	<1	
Exp. 3					<Plant extracts>						
					*Angelica shikokiana* Makino root						
		Day 0	10%	Aβ_1–42_	Leaf PBS extract	1472	μg/mL	417	μg/mL	3.5	[38]
					Root PBS extract	2686	μg/mL	107	μg/mL	25.1	[38]
					Seed PBS extract	2118	μg/mL	41.7	μg/mL	50.8	[38]
					Leaf NaHCO_3_ extract	1827	μg/mL	4.71	μg/mL	387.9	[38]
					Root NaHCO_3_ extract	2691	μg/mL	43.8	μg/mL	61.4	[38]
					Seed NaHCO_3_ extract	2441	μg/mL	73.9	μg/mL	33	[38]
		Day 6	1%	Paclitaxel	Root PBS extract	>2000	μg/mL	107	μg/mL	>18.7	[38]
											
		Day 6		Aβ_1–42_	Miso extract	6.83	%	0.039	%	175.1	[39]
		Day 6		Aβ_25–35_	Miso extract	6.83	%	0.191	%	35.8	[39]
Exp. 4					<Antioxidants>						
		Day 5		Paclitaxel	Docosahexaenoic acid	0.017		>0.02		<1	[35]
					Acetyl-l-carnitine hydrochlorid	>10		0.07		142.9	[35]
					*N*-Acetyl-l-cysteine	>10		2.25		>4.4	[35]
					Sodium ascorbate	0.52		>1		<1	[35]
